# Donor-Derived Infections: A Journey From Evidence to Policy

**DOI:** 10.1093/ofid/ofae670

**Published:** 2024-11-12

**Authors:** Vivek B Beechar, Stephanie M Pouch, Ricardo M La Hoz

**Affiliations:** Division of Infectious Diseases and Geographic Medicine, The University of Texas Southwestern Medical Center, Dallas, Texas, USA; Division of Infectious Diseases, Department of Medicine, Emory University School of Medicine, Atlanta, Georgia, USA; Division of Infectious Diseases and Geographic Medicine, The University of Texas Southwestern Medical Center, Dallas, Texas, USA

## Abstract

The prevalence of donor-derived Bartonella quintana infection poses an emerging issue in solid organ transplantation. Further studies are needed to validate criteria for donor screening to prevent transmission without negatively affecting organ utilization.

To the  Editor—The article “Donor-Derived *Bartonella quintana* Infection in Solid Organ Transplantation: An Emerging Public Health Issue With Diagnostic Challenges” by Boodman et al was recently published in *Open Forum Infectious Diseases* [1]. It highlights several cases of probable donor-derived transmission of *B quintana* infection in solid organ transplant recipients. Donor-derived infections carry a significant risk for morbidity and mortality, highlighting the importance of investigating potential preventative measures. Policy development is guided by evidence and also factors cost-effectiveness and feasibility. Herein, we review the fundamental principles for contextualizing the article findings when developing policy recommendations.

The authors refer to 8 cases of probable donor transmission events from 1 case series and 2 case reports [2–5]. The patients presented with a range of clinical syndromes, including bacillary angiomatosis, fever, rash, vertebral osteomyelitis, and endocarditis. These cases serve as the basis for proposing targeted donor screening for *B quintana* by using risk factor assessment (history of experiencing homelessness, etc) and physical examination findings (rashes, stigmata of endocarditis, etc). The proposed screening tests for *B quintana* in the donor include bacterial blood cultures (prolonged incubation), polymerase chain reaction (PCR) testing, and serology. The authors conclude with a scheme for recipients' posttransplantation monitoring within 1, 3, and 6 to 12 months, stratified by recipient risk category (high vs moderate risk) [1].

There are several limitations to the recommendations proposed by the authors. First and most important, the current level of evidence hampers our ability to draw definitive conclusions. Second, while the risk factors identified for screening donors are based on evidence-based epidemiologic studies, the authors acknowledge that obtaining such historical details for deceased organ donors may be challenging. Furthermore, the authors recognize the impracticality of expecting organ procurement teams to perform detailed examinations of every donor for signs of lice infestation, whether through skin examination or inspection of the donor's clothing [1]. Third, the available diagnostic tests—bacterial blood cultures with prolonged incubation, PCR testing, and serology—are not currently approved by the Food and Drug Administration for screening donors. Furthermore, the tests may not be readily available to organ procurement organizations, adding to the turn-around time [6]. These factors, particularly the first one, make it challenging to propose a policy change.

It is also important to note that there is no evidence that screening for *B quintana* via bacterial blood cultures, PCR testing, and serology improves transplant outcomes. All tests have false positives, which can be compounded by the number of tests performed. In addition, there may be unforeseen consequences on organ utilization when potential candidates or transplant programs evaluate an organ offer with multiple pending test results. Although this could be mitigated with education on how to proceed in such situations, the full impact of the proposed changes is unknown. In summary, will the suggested approach improve the system by preventing transmissions without affecting organ utilization? When potential policy change and its impact on system efficiency are being evaluated, assessment of the impact of nonutilized organs and candidates' wait-list mortality must be considered.

The Organ Procurement Transplant Network provides a 10-step framework for policy development that can guide approaching screening for emerging pathogens in donors [7]. The first step is to determine the magnitude of the problem—a more precise estimate of the incidence of donor-derived bartonellosis. An additional step will be to establish the optimal donor screening modality. The ideal approach would be easy to implement, have a rapid turnaround time, be cost-effective, and effectively prevent disease transmission without an impact on organ utilization. Pilot studies are needed to examine the effectiveness of using the screening regimen suggested by the authors. These studies are vital to developing a robust policy. The process of policy development also includes a public comment step, in which all the stakeholders of the transplant community assess the pros and cons. The input from organ procurement organizations is key when proposing changes in deceased donor testing. After incorporating the feedback, the finalized proposed policy needs to be approved by the Organ Procurement Transplant Network before implementation.

Recent studies [2–5] suggest an increased risk of donor-derived transmission of *B quintana* from donors who experienced homelessness. Current literature suggests that transplant physicians must maintain a high clinical index of suspicion for bartonellosis in symptomatic patients. Although the evidence does not yet justify targeted donor screening, the authors' call for further investigation into this issue is warranted.

## Notes

***Author contributions.*** All the authors have seen and approved the submission of this manuscript in its entirety. All the authors contributed significantly to the preparation of this manuscript.
